# Differences in Oxytocin Response Between a Group of Friends and a Group of Strangers Following Facilitated Drum Circle Activities

**DOI:** 10.1002/brb3.71183

**Published:** 2026-01-28

**Authors:** Mitsuru Kikuchi, Sanae Tanaka, Kazumi Furuhara, Haruhiro Higashida, Chiharu Tsuji

**Affiliations:** ^1^ Research Center for Child Mental Development Kanazawa University Kanazawa Japan; ^2^ United Graduate School of Child Development Osaka University, Kanazawa University, Hamamatsu University School of Medicine, Chiba University and University of Fukui Kanazawa Japan; ^3^ Department of Psychiatry and Neurobiology, Graduate School of Medical Science Kanazawa University Kanazawa Japan

**Keywords:** oxytocin, cortisol, group activity, drumming, saliva, workshop

## Abstract

**Background:**

Participation in arts‐related activities has been shown to positively influence the well‐being and social connectedness of young people. However, few studies have explored the neuroendocrinological changes that might contribute to these benefits. In this exploratory study, we assessed oxytocin (OT) and cortisol (CORT) responses in children experiencing facilitated drum circle activities for the first time. These activities involve imitation and coordinated movement, which are known to increase OT levels. Additionally, OT levels are associated with behavioral synchrony and affiliative behaviors in close preexisting relationships, with higher baseline levels observed in individuals who exhibit more affectionate and coordinated interactions. We hypothesized that children participating in facilitated drum circles with their friends would show higher levels of OT than those participating with strangers.

**Methods:**

Elementary school girls were assigned to either a friends group (F‐group) or a strangers group (S‐group). Salivary samples were collected before and after the facilitated drum circle activities.

**Results:**

Salivary OT increased after the activity in the F‐group but not in the S‐group. Salivary CORT showed no statistical difference between or within groups.

**Conclusion:**

Our current data suggest that participation in facilitated drum circles with friends may lead to an increase in OT levels in children, and that preexisting bonds may influence the neuroendocrinological response.

## Graphical abstract

Elementary school girls participated in a facilitated drum circle with either friends or strangers. Participation with friends increased salivary oxytocin, whereas no increase occurred with strangers. Cortisol levels did not differ between groups. Preexisting social bonds may influence oxytocin responses during facilitated drum circle participation.

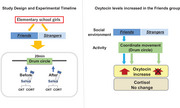

## 1 Introduction

Active engagement and collaboration in arts‐based activities among young people are known to have a positive impact on their well‐being and social connectedness (Williams et al. 2023). Numerous studies have documented positive effects on emotions and behaviors, including reduced stress, improved mood, and decreased anxiety (Williams et al. 2023). Group drumming is a popular and engaging activity that encourages active participation and collaboration. In studies using feedback and psychosocial questionnaires, group drumming has been shown to positively affect psychological states and well‐being (Ascenso et al. 2018). For instance, an exploratory study examining the psychological effects of 10 weeks of group drumming among health service users in the United Kingdom showed improvements in depression, anxiety, and social resilience (Fancourt et al. 2016). Meanwhile, studies of drum circles as a form of treatment for addiction in the United States of America reported that such drumming promotes relaxation, reduces feelings of alienation, and enhances a sense of connectedness with oneself and others (Winkelman 2003, Winkelman 2005). One notable program, the DRUMBEAT (Discovering Relationships Using Music—Beliefs, Emotions, Attitudes & Thoughts) program in Australia, combines musical experience with cognitive behavioral therapy to engage at‐risk youth (Faulkner et al. 2012). Hand drumming in the form of a drum circle is reported to yield positive outcomes, such as increased self‐confidence, improved relationships, and decreased school absenteeism (Wood et al. 2013). Overall, the above findings suggest that group drumming provides multifaceted benefits, including mental health improvements and enhanced social outcomes. However, the neuroendocrinological changes that might contribute to these outcomes have not been well studied.

Well‐being refers to being physically healthy and emotionally fulfilled, both within ourselves and in our relationships with others and our environment (Fancourt and Finn 2019). Positive interactions with the people around us and satisfaction in our relationships promote a sense of well‐being (Fancourt and Finn 2019). Social cohesion, social connectedness, and the ability to relate to others are aspects that contribute to well‐being and can potentially be influenced by OT (Ishak et al. 2011). OT is a neuropeptide that plays a pivotal role in social recognition and affiliative behaviors in mammalian species (Jurek and Neumann 2018, Andari et al. 2018). In humans, OT fosters prosocial behaviors such as trust, cooperation, and empathy in a context‐dependent manner, often favoring in‐group members (Andari et al. 2018, Bartz et al. 2011, Ross and Young 2009, Yao and Kendrick 2025). It also modulates affiliative behaviors, including the recognition of facial expressions and bonding (Andari et al. 2018, Ross and Young 2009). Further, OT is involved in the stress response, as it reduces anxiety (Andari et al. 2018, Neumann and Slattery 2016). By encouraging prosocial and affiliative behavior and fostering connections with others, OT may ultimately lead to emotional fulfillment and contribute to sustainable emotional well‐being (Ishak et al. 2011). Against this background, it can be anticipated that the emotional outcome of participation in a drum circle may be influenced by OT. However, no studies have yet examined the changes in OT levels specifically during drum circle activities, and the triggers of such changes in this context remain unknown.

While many drum circle activities involve elements of mimicry and rhythmic synchrony, facilitated drum circles are particularly structured to enhance these components. They are led by a trained facilitator who guides the group through coordinated drumming patterns to encourage interaction and group cohesion. The consequences of mimicry and/or synchronization and the outcomes of being administered OT share some similarities. Mimicry induces prosociality and increases interpersonal liking, rapport, and empathy (Chartrand and Van Baaren 2009). These behavioral and emotional effects are observed in both the mimicker and the person being mimicked. For instance, prosocial behavior is elicited in both the mimicker and the person being mimicked (Van Baaren et al. 2004, Müller et al. 2012). Infants were more likely to show altruistic behavior toward a female experimenter after being bounced to music in synchrony with her, compared with infants who were bounced asynchronously (Cirelli et al. 2014). Meanwhile, in another study examining how participants evaluated experimenters under synchronous and asynchronous conditions, participants rated the experimenter significantly more favorably in the synchronous condition (Hove and Risen 2009). Building on these findings, Spengler et al. demonstrated that synchronized social interaction, such as imitating hand gestures in dyadic situations, led to increased salivary OT levels in both the imitator and the person being imitated (2017). Reciprocal interaction within bonded relationships, such as between parents and their infants, induces OT release in the infant, and the degree of OT system reactivity in parent and child is interrelated (Feldman et al. 2010a). Therefore, OT release may be stimulated by mimicking and coordinated movements that occur during participation in a drum circle in both bonded and unbonded participants, although higher OT release may occur in the former.

In this exploratory study, we assessed the salivary levels of two hormones, OT and CORT, in elementary school girls who participated in facilitated drum circles for the first time. The recruited participants were assigned to two distinct groups: bonded (Friends group, F‐group) and unbonded (Strangers group, S‐group). We hypothesized that children who participate in such circles with friends would show higher OT levels than those participating with strangers. However, an increase in OT was detected only in the F‐group and not in the S‐group. Meanwhile, CORT levels showed no statistically significant differences across all time points within and between the two groups. We later discuss the difference between the initial hypothesis and the obtained results through the framework of the priming theory of OT (Ludwig and Leng 2006).

## 2 Methods

### 2.1 Subjects

**FIGURE 1:**
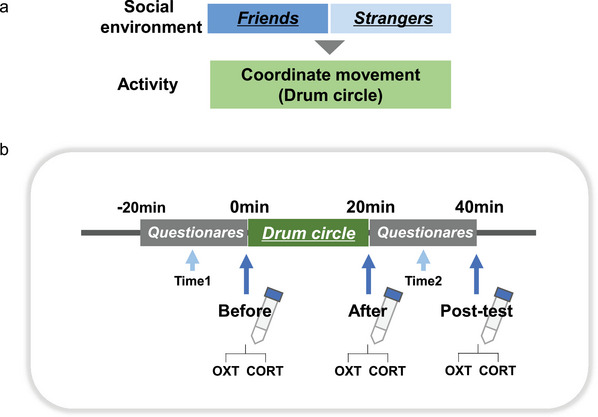
Scheme of this study. **(a)** Elementary school girls were recruited and allocated to two groups to participate in a facilitated drum circle. One group consisted of friends (F‐group), while the other consisted of strangers (S‐group) and **(b)** Timeline of the experiment including saliva sample collection points and questionnaires. Abbreviations: OXT, oxytocin; CORT, cortisol.

Typically developing girls aged 9–10 years, attending an elementary school, and with no prior experience of drum circle activities were recruited and allocated into two groups. One group comprised girls who did not know each other (Strangers group, S‐group), and the other comprised girls who were friends (Friends group, F‐group; Figure 1a). Facilitated drum circle activities were held with the S‐group three times, with either 7 or 4 participants. Similarly, such activities were held with the F group four times, with 3–5 individuals in attendance, each time with a different set of girls. For inclusion in the F‐group, all participants in each set had to meet the following criteria: (1) attending the same school and (2) playing together outside school hours. Additionally, each set of participants had further connections, either through having been in the same class for at least a year or by attending the same after‐school club continuously from the first grade throughout the year, including summer, winter, and spring holidays. All group members also spent time together occasionally on weekends.

### 2.2 Questionnaire

The parents of the participants were sent questionnaires—namely, the kid‐KINDL Japanese version for elementary and junior high school students (Ravens‐Sieberer 1998, Ravens‐Sieberer and Bullinger 1998, Furusho et al. 2014) and the Japanese version of the Social Responsiveness Scale‐Second Edition (SRS‐2) (Constantino 2009, Kamio et al. 2013)—and were asked to complete them at home regarding their children. The Kid‐KINDL is a questionnaire that measures children's quality of life. The SRS‐2 measures the severity of autistic symptomatology as a quantitative trait among children clinically affected by autism spectrum conditions, as well as among children in the general population. On the day of the facilitated drum circle, the children brought the questionnaires completed by their parents. Before the activity began, the children completed the self‐reported version of Kid‐KINDL, the Japanese version of the Spence Children's Anxiety Scale (SCAS) (Spence 1998), and the Depression Self‐Rating Scale for Children (DSRS‐C) (Birleson 2011). Demographic variables, including age, did not show significant differences between the groups, as shown in Table 1.

**TABLE 1:** Age and psychological assessment scores of participants who underwent salivary data analysis.

	All	Friends	Strangers	Friends/Strangers
	Mean (SD) *N* = 28	Mean (SD) *N* = 14	Mean (SD) *N* = 14	*p*‐value (Mann–Whitney *U)*
**Month of age**	120.1 (4.93)	121.6 (3.92)	118.6 (5.51)	0.095 (61.5)
**SCAS**	25.1 (13.16)	23.43 (9.49)	26.79 (16.23)	0.708 (89.5)
**DSRS‐C**	8.46 (4.75)	8.64 (5.27)	8.29 (4.36)	0.955 (96.5)
**SRS‐2**	47.00 (10.55)*	47.33 (9.23)^†^	46.71 (11.91)	0.693 (76.0)
**KINDL** **(Children)**	72.62 (13.19)	69.79 (15.25)	75.45 (10.57)	0.345 (77.0)
**KINDL** **(Parents)**	78.53 (9.52)	78.13 (10.04)^†^	78.87 (9.42)	0.990 (83.5)

^*^ *N* = 2 6 ^†^ *N* = 12.

Abbreviations: DSRS‐C, Depression Self‐Rating Scale for Children; KINDL, Questionnaire for Measuring Health‐Related Quality of Life in Children and Adolescents; SCAS, Spence Children's Anxiety Scale; SRS‐2, Social Responsiveness Scale Second Edition.

The emotional state on the day of the facilitated drum circle was assessed using a questionnaire with a Visual Analog Scale (VAS). For this scale, vertical lines 100 mm in length were presented, with anchor statements at the top and bottom. Before and after the workshop, participants were asked questions about their happiness, vitality, enjoyment of the arts activity, and relaxation. After the workshop, they were also asked how they felt about participating in the facilitated drum circle, including whether they were satisfied, had fun, or if the activity had become their favorite. Higher scores indicate more positive responses.

### 2.3 Ethics Statement

This study was approved as a noninvasive medical study by Medical Ethics Committee of Kanazawa University in 2018 (approval number #2018‐025). It was conducted in accordance with the Ethical Guidelines for Clinical Studies of the Ministry of Health, Labour and Welfare of Japan and the tenets of the Declaration of Helsinki. Both participants and their parents were given a complete explanation and written information about the study, and both subsequently provided written informed consent. On the day of the facilitated drum circle, participants were reminded that they could choose to withdraw from the study at any time or opt out of saliva sampling, even after agreeing to participate.

### 2.4 The Flow of the Experiments

The participants arrived 30 min before the facilitated drum circle. The facilitated drum circle activities started at 10:30 a.m., except for one S‐group session, which was held at 2:30 p.m. Upon arrival, the participants were seated together in the same room, received an explanation of the workshop, and completed the questionnaires until the start of the drum circle activity. Saliva sampling was performed just before the participants moved to sit in front of the drums. After 20 min of facilitated drum circle activities, they returned to their previous seats and immediately underwent a second saliva sampling. Next, while waiting a further 20 min for the final saliva sample to be taken, they completed the questionnaires. When they had finished the questionnaires, they were given a *Spot the Difference* worksheet to complete until the final saliva sampling. From their arrival until the end of the experiment, the participants had no contact with their parents or guardians.

### 2.5 Activities of the Facilitated Drum Circle

The drum circle was based solely on drumming, without singing or background music. Drums were placed in a circle, and the children chose a drum they liked and sat by it. The same facilitator (first author, licensed) and assistant were present at every workshop. They were positioned among the participants with the drums, although the facilitator occasionally moved to the center to lead the children. The facilitator had no prior acquaintance with the participants and was blinded to the group classifications throughout the session. Examples of drum activities included “Call and Response,” “Drum Circle Freeze,” and “Drum Jam.” The facilitator was certified as a trained facilitator through Beat of Success (http://bos1.jp; Ochiai, Tokyo, Japan). This organization is a full member of the Drum Circle Facilitators Association (https://dcfa.jp/, Ginza, Tokyo, Japan), which is authorized by REMO Inc. (Valencia, CA, USA), a United States instrument manufacturer that promotes and supports drum circle activities worldwide.

### 2.6 Saliva Collection

Participants were instructed to avoid eating, drinking anything other than water, brushing their teeth, or engaging in any physical activities for at least 1 h before attending the activities. The participants arrived 30 min before the workshop started. Upon arrival, they were first asked to drink some water if needed, but were then instructed to avoid drinking any further water until after the final saliva sampling. Saliva samples were collected at three time points: just before the start of the drum circle, immediately after the 20‐min drum circle activity, and 20 min after the activity had finished. The saliva samples (1.0–1.5 mL) were collected in sterile 15 mL polypropylene tubes (Greiner Bio‐one Co. Ltd., Tokyo, Japan) and immediately placed on ice. After the participants were dismissed, the samples were stored at −20°C.

### 2.7 Saliva Measurement

The saliva samples stored at −20°C were thawed and then centrifuged at 4°C and 1,500 × g for 10 min. The samples were divided into 1.5 mL microtubes, each containing ≥ 100 µL, and kept at −80°C until assaying. Salivary OT levels were measured using a 96‐well plate commercial OT‐ELISA kit (Enzo Life Sciences, Farmingdale, NY, USA), as previously described (Tanaka et al. 2020, Yuhi et al. 2017, Minami et al. 2022, Sugiyama et al. 2024). Measurements were performed in duplicate. Samples (100 µL), without purification, were treated according to the manufacturer's instructions. The optical density of the samples and standards was measured at a wavelength of 405 nm using a microplate reader (Bio‐Rad, Richmond, CA, USA). Salivary CORT levels were measured using a CORT enzyme immunoassay kit (Salimetrics, State College, PA, USA), as previously described (Sugiyama et al. 2024, Yuhi et al. 2018). In this case, 25 µL samples were treated according to the manufacturer's instructions. The optical density of the samples and standards was measured at a wavelength of 450 nm using the same microplate reader. All measurements were performed in duplicate. Sample concentrations were calculated based on the relevant standard curve. The intra‐ and inter‐assay coefficients were <7.8% and <8.2%, respectively. Samples were coded, and the experimenter responsible for saliva measurement was blinded to the participants’ group allocation.

### 2.8 Data Analysis

In this study, we considered the potential impact of circadian rhythm on the facilitated drum circles held in the afternoon. However, because we did not observe a significant reduction in CORT levels during 90‐min visual art workshops in typically developing 10‐year‐old children in either the morning (unpublished data) or afternoon (Tanaka et al. 2020) sessions, we pooled the saliva data from the morning and afternoon S‐group drum circle sessions. For data analysis, three girls in the S‐group session with seven participants were excluded, as it was later discovered that they knew each other. Additionally, the CORT data of another S‐group participant were excluded because her level was identified as an outlier using Prism 8 software (GraphPad Software Inc., San Diego, CA, USA).

### 2.9 Statistical Analysis

All statistical analyses were performed using Prism 9.3.1 software (GraphPad Software Inc., San Diego, CA, USA). Outliers were identified using the robust regression and outlier removal (ROUT) method implemented in GraphPad Prism, with the false discovery rate (*Q*) set at 1%. The relative values of salivary OT and CORT at each time point were calculated by dividing the value at that time point by the pre‐workshop baseline level. Since the absolute values of OT were not normally distributed, they underwent a log10 transformation. Log10‐transformed values were used for statistical analysis and data visualization. Statistical analysis was conducted using two‐way repeated measures ANOVA, with Bonferroni's multiple comparison test used for post hoc analysis. As the absolute values of CORT before activity in the S‐group were also not normally distributed, the Mann–Whitney U test and the Wilcoxon signed‐rank test were used to assess differences both between and within groups. Post hoc statistical power analysis was conducted using G*Power 3.1 (Faul et al. 2007). All data are presented as mean ± SEM. In all analyses, *p* < 0.05 was considered statistically significant.

## 3 Results

**FIGURE 2:**
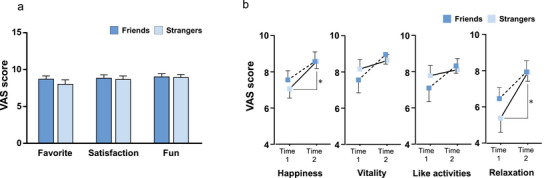
Emotional state measured with the visual analog scale (VAS). (**a**) Participants’ responses after the facilitated drum circle. No significant difference was detected between groups and (**b**) Emotional changes were measured using four questions with VAS before and after the facilitated drum circle. Higher VAS scores indicate a more positive response. Data are mean ± SEM. *p < 0.05. Friends, *n* = 14; Strangers, *n* = 14.

Saliva samples were collected from groups of girls who were either friends (F‐group) or strangers (S‐group). Sampling was performed before, immediately after, and 20 min after the facilitated drum circle (Figure 1). We also assessed the girls’ emotions via a questionnaire using the VAS (Figure 2). Scores were relatively high for both groups, with no significant differences in the VAS scores for the questions asking how they felt about the facilitated drum circle after participating in it (Figure 2a). However, when comparing the emotional states measured by the questionnaire before (time 1) and after (time 2) the facilitated drum circle, while the F‐group reported no change, the S‐group reported significantly higher happiness (Student's *t*‐test, *p* = 0.034) and relaxation (Student's *t*‐test, *p* = 0.012) scores at time 2 (Figure 2b).

**FIGURE 3:**
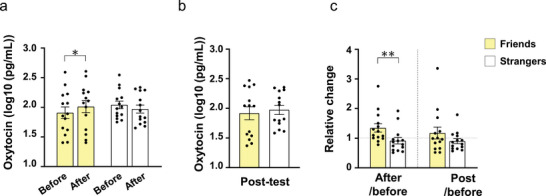
Changes in salivary oxytocin levels during facilitated drum circle activities. (**a**) Absolute salivary oxytocin concentration before and immediately after the facilitated drum circle, (**b**) Absolute salivary oxytocin concentration of the Friends and Strangers groups 20 min after the facilitated drum circle, and (**c**) Relative changes in salivary oxytocin concentration immediately and 20 min after the facilitated drum circle. Oxytocin concentrations are shown as log10‐transformed values. Data are mean ± SEM. **p < 0.01. Friends, *n* = 14; Strangers, *n* = 14.

The salivary OT level before participating in the facilitated drum circle was similar between the F‐ and S‐groups. In the F‐group, the OT level increased following the facilitated drum circle (Figure 3a). A two‐way repeated measures ANOVA was conducted to evaluate differences in OT concentration between two time points—before and after the activity. There were no significant main effects for either time (*F* [1, 26] = 0.301, 𝜂^2^p = 0.011, *p* = 0.588) or group (*F* [1, 26] = 0.1575, 𝜂^2^p = 0.083, *p* = 0.695). However, there was a significant interaction between time and group (*F* [1, 26] = 8.268, 𝜂^2^p = 0.241, *p* = 0.008; Figure 3a). A post hoc power analysis for the interaction effect (time × group) yielded a Cohen's f of 0.564 and a power of approximately 0.80 at α = 0.05, indicating that the analysis was adequately powered to detect the observed effect. Subsequent post hoc analysis by Bonferroni's test demonstrated a significant increase in OT concentration after the facilitated drum circle activity in the F‐group (*p* = 0.046), but not in the S‐group (*p* = 0.224). There was no difference in OT concentration between the two groups 20 min after the drum circle activity (Figure 3b). The relative change in OT concentration immediately after the drum circle showed significant differences between the F‐ and S‐groups (Mann–Whitney, *p* = 0.005), but no difference 20 min after the drum circle (Figure 3c).

**FIGURE 4:**
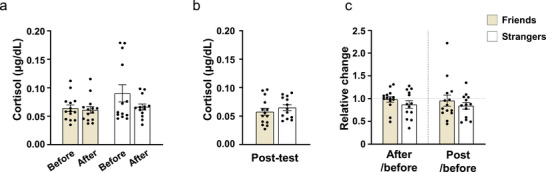
Changes in salivary cortisol levels during facilitated drum circle activities. (**a**) Absolute salivary cortisol concentration before and immediately after the facilitated drum circle, (**b**) Absolute salivary cortisol concentration of the Friends and Strangers groups 20 min after the facilitated drum circle, and (**c**) Relative changes in salivary cortisol concentration immediately and 20 min after the facilitated drum circle. Data are mean ± SEM. Friends, *n* = 14; Strangers, *n* = 13.

Although some cortisol values in the S‐group were notably higher than the rest of the dataset before the facilitated drum circle activity, statistical analysis revealed no significant differences in CORT levels between the F‐ and S‐groups, either before or after the activity (Figure 4a). Moreover, cortisol levels remained stable within each group across time points, and both groups exhibited comparable levels 20 min after the activity (Figure 4b). The relative change in CORT concentration immediately after and 20 min post‐drum circle showed no difference between groups (Figure 4c).

## 4 Discussion

An increase in salivary OT was observed exclusively in the F‐group after the facilitated drum circle session. Since the imitation and coordinated activities were nearly identical for both the F‐group (friends) and the S‐group (strangers), one would expect both groups—those in which friends performed drumming together (F‐group) and those where strangers did so (S‐group)—to show an increase in salivary OT levels. Furthermore, previous research has shown that reciprocal interaction within bonded relationships, such as between parents and their infants, induces the infant's OT level, and the degree of reactivity of the OT system in parent and child is interrelated (Feldman et al. 2010). Therefore, the initial hypothesis was that closer social bonds, like those in the F‐group, would result in a more pronounced increase in salivary OT levels. Yet, this was not the case, suggesting that social familiarity plays a role in OT increase during shared rhythmic experiences, beyond the physical movements themselves. OT is known to mediate various physiological and social processes; however, the specific conditions that most significantly influence its level in real‐life settings are not yet fully understood. It can be said that examining salivary OT levels under naturalistic conditions was essential for advancing our understanding of its role in everyday human interactions and social behaviors.

Although the overall CORT levels did not show a statistically significant difference, it is noteworthy that some individuals in the S‐group exhibited markedly elevated CORT levels prior to the activity. This variability underscores the complexity of the stress response system and may reflect individual differences in sensitivity to novel environments. The difference in CORT levels may have been attributable to their response to the unfamiliar environment, whether faced with or without familiar peers. Social supports are known to dampen the stress response (Cohen and Wills 1985). Adams et al. reported that, when a best friend was present, there was less change in CORT due to the negativity of an experience (Adams et al. 2011). Meanwhile, in children aged 9–10 who underwent a modified Trier Social Stress Test with support from their parents, the CORT stress response was significantly reduced compared with that of subjects without such support (Hostinar et al. 2015). Therefore, social support from peers could have mitigated the stress response and influenced the salivary CORT level in the F‐group, preventing the occurrence of excessively high salivary CORT levels observed in some individuals in the S‐group. Taken together, these findings suggest that the OT increase observed in the F‐group is unlikely to reflect aversive physiological stress but rather social regulatory processes engaged in response to social stimulation, which may occur independently of measurable CORT elevation.

In the present study, we observed a significant increase in salivary OT concentrations following a facilitated group drumming activity, but only among participants in the friend group. Previous research by Feldman et al. reported that mothers who engaged in more affectionate behaviors toward their infants had higher baseline levels of OT, suggesting a potential association between OT and affiliative behavior (2010). Further, De Dreu et al. showed that intranasal OT administration selectively increased prosocial behaviors—such as trust and cooperation—for in‐group members, but not for out‐group members (2010). Although the present study does not directly replicate De Dreu et al.’s experimental design, as it did not involve exogenous OT administration followed by behavioral assessment, our finding of an in‐group‐specific increase in endogenous OT may nonetheless reflect a similar relational specificity within the OTergic system. Future research is needed to determine whether such OT responses are linked to social relationships in comparable activities in children.

**FIGURE 5:**
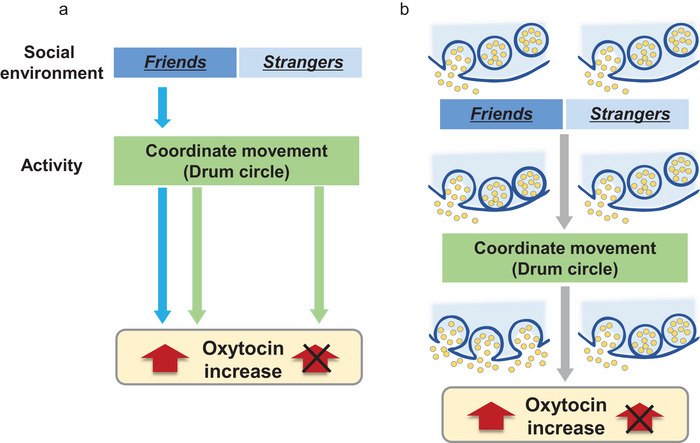
Graphical summary. (**a**) Salivary oxytocin and cortisol concentrations in typically developing children girls participating in facilitated drum circle activities with friends or strangers were measured. An increase in oxytocin was observed only in participants with friends. Preexisting bonding states may influence neuroendocrinological responses to novel social activities such as facilitated drum circles and (**b**) The priming theory may provide a useful framework for understanding the differential oxytocin responses observed. In girls with friends, initial interaction stimulates oxytocin vesicles to move closer to the membrane, followed by coordinated movement triggering oxytocin release. In contrast, in girls with strangers, while coordinated movement relocates the vesicles closer to the membrane, no oxytocin release is observed at this moment.

An increase in OT levels after the facilitated drum circle was observed in the F‐group but not in the S‐group (Figure 5a). Although the mechanism for the difference in OT response among groups needs further exploration, one possible explanation may be better understood through the framework of the priming theory (Ludwig and Leng 2006). This theory has been proposed to explain the mechanism of dendritic OT release. Simply put, with the first exposure to a stimulus, dense core vesicles of OT transition into a “ready‐to‐release” state, moving closer to the cellular membrane. Upon subsequent stimuli, these primed vesicles release OT in response. While it remains unclear whether this neuronal mechanism directly applies to the human brain or periphery, the concept of sequential stimuli in the priming theory may provide a useful viewpoint for interpreting our observed phenomenon (Figure 5b). For the F‐group, these sequential stimuli—first, pre‐existing social bonds, and second, the facilitated drum circle activity—appear to work in tandem to enhance OT release. The presence of social bonds may have primed the OT system, preparing it for release. Subsequently, the facilitated drum circle activity, characterized by physical coordination and social interaction, acted as a trigger to facilitate OT release. In contrast, the non‐friends group may lack the initial priming stimulus of social bonds, which could explain the absence of significant OT release during the activity. While synchronized movements might stimulate their OT system over time, repeated interactions or additional exposure would likely be needed to achieve a similar “ready‐to‐release” state. These findings may highlight the importance of social context and sequential stimulation in modulating OT dynamics. Although we do not claim that the molecular mechanism of priming theory directly applies in this case, its conceptual framework—centered on the role of sequential events—offers a useful perspective on how pre‐existing social bonds and shared activities may jointly enhance OT release.

In contrast to the current results, our group previously reported on salivary OT levels during Japanese drumming among maltreated children (Yuhi et al. 2017). The salivary OT level was examined before and after the practice session aimed at preparing for a future performance in front of an audience and at the actual performance (Yuhi et al. 2017). In this context, although the activity involved synchronous movement, the drumming was not a simple, enjoyable reciprocal activity for the children. OT levels showed mean changes, but no statistically significant increase was detected in boys from elementary to junior high school during drumming practice and performance (Yuhi et al. 2017). While activities involving music are generally considered to be a pleasant experience, this is not always true in every situation. For instance, listening to or playing certain pieces of music associated with unpleasant memories can trigger feelings of melancholy or worsen negative emotions (Hays and Minichiello 2005). In line with this, Steptoe reported that performing music can induce negative emotions and increase feelings of anxiety (Steptoe 2001). Although CORT levels were not assessed in our previous study (Yuhi et al. 2017), the maltreated children may have experienced elevated CORT levels in response to Japanese drumming. Comparing the current and previous results, the divergent purposes and circumstances of the group drumming may have resulted in different OT‐related outcomes.

Our current study had several limitations. First, the number of subjects was small, and only girls were examined. The susceptibility to a new environment or the role of social support may differ between the sexes. As such, future work should examine larger samples and include an age‐matched group of boys. Second, we examined two groups of girls: one consisting of friends and the other of strangers. Each subject participated only once in either the F‐group or the S‐group, but not in both. To rigorously assess the effects of in‐group and out‐group membership, the same participants would ideally experience both conditions. However, owing to the limited number of participants, our design required that each girl participate in only one group condition. Further research is thus needed to thoroughly examine the effects of in‐group and out‐group status with more participants. Third, in our experiment, participants were not separated from each other while waiting for the drum circle to start. The participants’ anxiety levels toward the new environment may thus have differed based on the social support they received during this waiting time. Therefore, we may not have been able to purely capture how the presence of friends influences changes in salivary OT levels during facilitated drum circle activities. Further experiments are also needed to determine the correlation between the neuroendocrinological changes that occurred during the facilitated drum circle activities and the participation of supportive members. Fourth, in our studies, we did not measure the degree of friendship or bonding among the children using questionnaires for the girls in the F‐group. Further experiments should be performed to determine the correlation between the neuroendocrinological response during the facilitated drum circle activities and the degree of bonded relationships. Finally, we used ELISA to measure the salivary OT concentrations. Although we consistently found this concentration to be within a similar range in our studies, this range was higher than that reported elsewhere (Schladt et al. 2017, Feldman et al. 2010). This may be attributable to the technical limitations of ELISA, including issues regarding antibody specificity. The antibody used in the kit may detect not only the free form of the target analyte but also different forms such as precursors, metabolites, or forms within other protein complexes (Yamamoto and Higashida 2020, Yamamoto et al. 2025, Carter et al. 2020). Although these factors may partially explain the differences in absolute values observed between different ELISA kits and between other methods such as HPLC‐MS or radioimmunoassay, we were still able to detect physiological changes in peripheral OT concentration during pregnancy, labor, and/or lactation using ELISA in blood and saliva samples (Minami et al. 2022). Therefore, while the values may not represent absolute physiological levels of free OT, the within‐subject changes are likely reliable. In addition, there is still ongoing discussion about whether peripheral OT levels reflect systemic or brain OT levels (Kagerbauer et al. 2013). However, a correlation between cerebrospinal fluid and peripheral OT concentration has been demonstrated in several studies (Martin et al. 2018, Carson et al. 2015). In the current study with young participants, saliva sampling was particularly useful.

The World Health Organization summarized the role of arts activities and interventions in improving the health and well‐being of both healthy individuals and those experiencing mental health challenges (Fancourt and Finn 2019). It also emphasized the importance of verifying and evaluating the effectiveness of participation in arts activities (Fancourt and Finn 2019). Our study focused on capturing fluctuations in neuroendocrine responses during music‐related activities. The current study with neuroendocrinological data may provide supportive evidence that activities such as drum circles can bring benefits to health and well‐being. By integrating our physiological measurements with psychological, emotional, or behavioral assessments, our study offers the advantage of quantitatively validating the effectiveness of arts activities. In the future, we plan to investigate the beneficial effects of these workshops on both healthy individuals and those experiencing mental health challenges. Furthermore, we intend to expand our study to investigate the association between neuroendocrine responses, qualitative measurements, and participant traits to better understand the effectiveness of participating in arts‐related activities for different individuals and in various aspects.

## 5 Conclusion

In conclusion, this pilot experiment aimed to measure salivary OT and CORT concentrations in typically developing children participating in facilitated drum circle activities. Our preliminary data suggest that participation in such circles may elicit OT release in young children. In addition, it revealed that preexisting social bonds may influence the neuroendocrinological response during new experiences. This study highlights the potential positive benefits of participation in facilitated drum circles and underscores the importance of social bonding and social context for reaping these benefits.

## Author Contributions

**Mitsuru Kikuchi**: investigation (facilitator of facilitated drum circle), resources, methodology, funding acquisition, and manuscript review and editing. **Sanae Tanaka**: investigation, methodology, funding acquisition, and manuscript review and editing. **Kazumi Furuhara**: investigation and data analysis. **Haruhiro Higashida**: methodology, manuscript review, and editing. **Chiharu Tsuji**: investigation, methodology, data analysis, validation, writing an original draft, manuscript review, and editing.

## Declaration of Generative AI in Scientific Writing

The authors declare that generative AI tools were used for grammar checking and proofreading and not for generating any scientific content. The OpenAI Large Language Model (LLM) ChatGPT version GPT‐5.1 was used for these purposes.

## Funding

This research was supported by the Moonshot Research and Development Program (grant number JPMJMS229C) from the Japan Science and Technology Agency (JST).

## Conflicts of Interest

The authors declare no competing interests.

## Data Availability

The data that support the findings of this study are available from the corresponding author upon reasonable request.
